# The urban desirability paradox: U.K. urban-rural differences in well-being, social satisfaction, and economic satisfaction

**DOI:** 10.1126/sciadv.adn1636

**Published:** 2024-07-19

**Authors:** Adam Finnemann, Karoline Huth, Denny Borsboom, Sacha Epskamp, Han van der Maas

**Affiliations:** ^1^ University of Amsterdam, Nieuwe Achtergracht 166, 1018 WV Amsterdam, Netherlands.; ^2^ Centre for Urban Mental Health, Nieuwe Achtergracht 166, 1018 WV Amsterdam, Netherlands.; ^3^ Amsterdam University Medical Center, Meibergdreef 9, 1105 AZ Amsterdam, Netherlands.; ^4^ National University of Singapore, 21 Lower Kent Ridge Rd, Singapore 119077, Singapore.

## Abstract

As the majority of the global population resides in cities, it is imperative to understand urban well-being. While cities offer concentrated social and economic opportunities, the question arises whether these benefits translate to equitable levels of satisfaction in these domains. Using a robust and objective measure of urbanicity on a sample of 156,000 U.K. residents aged 40 and up, we find that urban living is associated with lower scores across seven dimensions of well-being, social satisfaction, and economic satisfaction. In addition, these scores exhibit greater variability within urban areas, revealing increased inequality. Last, we identify optimal distances in the hinterlands of cities with the highest satisfaction and the least variation. Our findings raise concern for the psychological well-being of urban residents and show the importance of nonlinear methods in urban research.

## Teaser

Despite the socioeconomic opportunities in cities, U.K. urban residents struggle with well-being and socioeconomic satisfaction.

## INTRODUCTION

The growth of cities is a hallmark of modernity and a central driver of its economic and technological acceleration (*1*, *2*). The percentage of individuals residing in cities has itself accelerated from 10% in the 1910s to a projected level of 68% by 2050 (*3*). This development means that cities are increasingly shaping our psychological lives, making it imperative to understand and tackle the risk factors associated with urban living.

Why are cities popular? Theories of urban agglomeration and urban scaling agree on an answer. Bringing people, companies, ideas, and technology in physical proximity generates synergies and with that tremendous wealth, innovation, creativity, and knowledge (*4*–*7*). Across countries, a 10% increase in urbanization is correlated with a 61% rise in per capita gross domestic product (*8*), and within countries, the world’s 23 megacities exceed the country’s average gross domestic product by 80% (*9*). Thus, urbanization has concentrated people and high-paying jobs in cities. The synergistic benefits are not limited to the economy but also generate rich cultural, social, and educational offerings (*10*). The urban popularity can therefore be explained in terms of the “urban promise” of social, cultural, educational, and economic opportunities (*11*).

A central question is whether cities live up to that promise. That is, do urban opportunities translate into a psychological advantage of increased subjective well-being that one might expect? The frequently studied area of urban-rural differences in happiness suggests a negative answer (*12*–*14*). For economically developed countries, rural areas consistently show higher happiness. A finding termed the “rural happiness paradox” in the economic literature. The sociological tradition, which portrays urban life as overwhelming, impersonal, and indifferent, likely finds this less paradoxical (*15*–*17*). This negative outlook is supported by recent empirical studies documenting urban disadvantages: Contagious diseases (*18*), air pollution (*18*, *19*), heat island effects (*20*), and poverty (*20*, *21*). Moreover, traffic problems scale with city size such that more severe congestion and commuting times are found in larger cities (*22*–*24*). Cities are also home to organized crime and elevated crime rates (*25*, *26*). Furthermore, the scarcity of green and blue spaces together with noise induces urban stress (*18*, *27*–*32*).

This host of stressors might impede overall urban happiness. Still, a defining feature of cities is their abundance of social and economic opportunities. Their abundance of social and economic opportunities suggests that urban residents might do well in terms of social and economic satisfactions. The existing literature on urban social and economic satisfactions is unfortunately sparse and conflicting. For economic satisfaction, one U.S. study reports that urban residents experience lower satisfaction compared to their rural counterparts (*33*). For social satisfaction, one study evidences increased social well-being in urban cores (*34*), while other studies have found no or opposite effects (*35*, *36*).

**Fig. 1.**
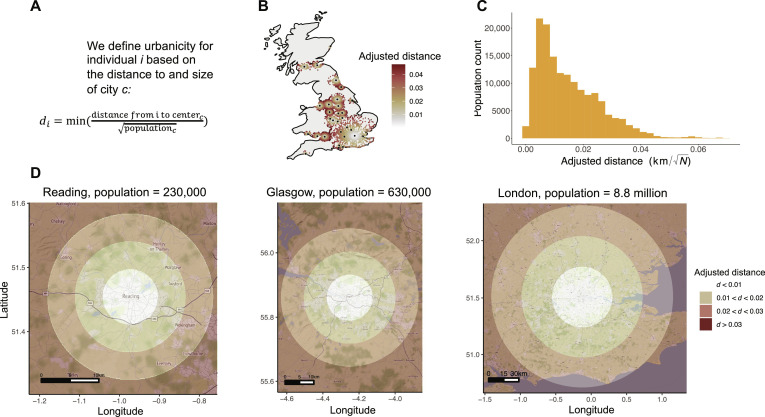
How we measure urbanicity. (**A**) The equation used to compute our urbanicity measure. (**B**) Map of the United Kingdom, highlighting city centers and coloring participants according to their urbanicity level. (**C**) Histogram of urbanicity scores. (**D**) Our measure has its minimum at city centers and increases linearly depending on the size of the city (we refer to it as a measure of “urbanicity,” although “rurality” might be more apt). We illustrate the urban gradient for three cities of different sizes.

Here, we aim to evaluate the urban promise by overcoming one methodological and conceptual limitation of the previous literature. Methodologically, the existing studies either rely on urban-rural boundaries or equate urbanicity with population density. Drawing boundaries is undesirable since statistical results are highly sensitive to how these boundaries are drawn (*37*). Thus, a continuous boundary-free definition is desirable (*38*). The most common continuous measure is population density [see, e.g., (*34*, *35*)], but it has been criticized for reducing cities to only one of the myriad factors that contribute to their complexity (*38*). Instead, we propose a geographic and continuous measure that is correlated with the myriad of urban factors. Our intuition is that city centers are highly urban and areas far from any city centers are highly rural. We operationalize this by defining the urbanicity of an individual as the distance to the nearest city center. Furthermore, we normalize this distance based on the size of the city to account for the difference between being, e.g., 10 km from London and Leeds—the former is a dense city, while the latter is countryside. This measure avoids drawing city boundaries and enables us to detect sub- and peri-urban effects through nonlinear statistical modeling (we explain our measure further in Fig. 1).

In addition to the methodological advance, we also bring a conceptual one. Inequality is arguably the most pervasive urban problem (*39*, *40*). Cities disproportionately favor the already favored, not only in wealth and income but also in health and social inequalities (*41*, *42*). This would suggest that the urban promise only benefits a minority of individuals. Unfortunately, little is known about the distribution of well-being, social satisfaction, and economic satisfaction in urban contexts.

In summary, it remains unclear to what extent the urban promise of socioeconomic opportunities translates into equitable levels of socioeconomic satisfaction. Here, we tackle this question using a large sample of 156,000 individuals aged 40 to 70 from the U.K. Biobank. Specifically, we explore two questions using our novel, continuous, and objective measure of urbanicity. First, how does the average well-being, as well as social satisfaction and economic satisfaction, vary across the urban-rural gradient? Second, how does the variation in the three psychological domains change with urbanicity?

## RESULTS

U.K. cities with populations exceeding 200,000 were included in the main study resulting in 28 city centers (see figs. S1 and S2 for analyses including smaller cities). Two cities, Middlesbrough and Oxford, were included despite having fewer than 200,000 inhabitants because these areas were highly sampled. Of the sampled participants, 157,116 (99.6%) lived within *d* < 0.7 of a city center and 155,279 lived within a radius of *d* < 0.05 (98.7%). The effective sample sizes varied considerably because of some questions being introduced into the U.K. Biobank at later stages of the data collection (Table 1). For well-being, 156,211 reported general happiness and 153,376 reported meaningful life. For the social variables, 155,150 participants completed the loneliness item, while 57,870 and 57,770 stated satisfactions with family and friendship. In the economic domain, 141,387 participants provided their income, while 42,389 and 58,127 provided satisfaction with their job and financial situation. The full sample had an average age of 55 (SD = 7.7), and 57% were female. The sample is 97% white, 0.8% Black, 0.9% Asian, and 1.4% other.

**Table 1.** Overview of included questions, number of responses (*N*), answer structure, and to which psychological dimension we assign the question (well-being, social, or economic variable).

Category	Answer type	*N*	Question
Well-being	Six-point Likert	156,211	In general, how happy are you?
Well-being	Six-point Likert	153,376	To what extent do you feel your life to be meaningful?
Social variable	Six-point Likert	57,870	In general, how satisfied are you with your family relationships?
Social variable	Six-point Likert	57,770	In general how satisfied are you with your friendships?
Social variable	Binary	155,150	Do you often feel lonely?
Economic variable	Five intervals (see text)	141,387	What is the average total income before tax received by your household?
Economic variable	Six-point Likert	58,127	In general how satisfied are you with your financial situation?
Economic variable	Six-point Likert	42,389	In general how satisfied are you with the work that you do?

### Mean level results

**Fig. 2.**
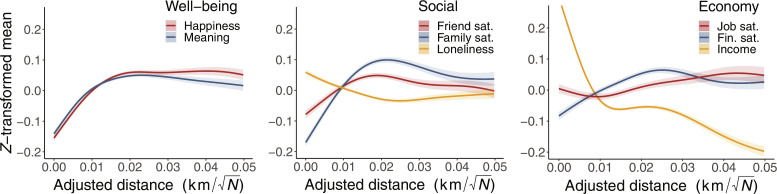
Urban gradients and 95% confidence bands show how means vary as a function of urbanicity for eight variables grouped along three psychological dimensions. Left: Well-being. Middle: Social satisfaction. Right: Economic satisfaction. sat., satisfaction; fin., financial. Lower values generally indicate unhealthy responses, except loneliness, for which lower values are healthier.

Figure 2 displays how mean levels change with urbanicity. The two well-being variables (left-most figure) show similar patterns across the urban gradients, with the lowest reported average for general happiness and meaningful life found closest to city centers. Both increase as we move from *d* = 0 to their maximum located around *d* = 0.018 [we analyze the location of the maxima (optimal distances) later]. From *d* = 0.018 to *d* = 0.05, both measures show slight declines. We also quantified the difference between the minimum value at city centers, *d* = 0, and the maximum value around *d* = 0.018. For general happiness, this change is 0.15 SD_happiness_, and for meaningful life, it is 0.19 SD_meaning_.

The middle plot of Fig. 2 reveals trends in our three social factors. We see the lowest average family and friendship satisfactions, as well as the highest loneliness, near city centers. Consequently, we consistently observe the poorest social satisfaction in cities across all three factors. We also see three optimal distances at intermediate radii around *d* = 0.02. At further distances from city centers, family and friendship satisfactions decline, while loneliness slightly increases. Again, we quantify the difference in estimated social satisfaction at *d* = 0 and the optimal distances. The difference in family satisfaction is 0.26 SD_family_; for friendship satisfaction, it is 0.12 SD_friendship_; and for loneliness, it is 0.09 SD_loneliness_.

Last, we explore the economic factors in the right-most plot of Fig. 2. We first focus on income and financial satisfaction, which show the largest changes—unexpectedly, in an inverse fashion. While we see a sharp increase in income near city centers, financial satisfaction steadily decreases from *d* ≈ 0.025 to *d* = 0. Again, we quantify the difference between their minimum and maximum values. Income changes by 0.51 SD_income_ across the urban gradient, while financial satisfaction decreases by 0.12 SD_finance_ from its maximum around *d* = 0.025 to *d* = 0. Last, we find an effect of urbanicity on job satisfaction, which increases in rural areas. The overall difference between its minimum and maximum is 0.08 SD_job_.

Figure S1 demonstrates how varying analysis choices affects the robustness of our results. Overall, our results are highly robust to including smaller cities, controlling for age and income, adjusting for participant bias, an alternative city size definition, using population density, excluding London, and analyzing different ethnic groups separately. The main exceptions are as follows: (i) Excluding London strongly diminishes the effect of urbanicity on income; (ii) controlling for income enhances the effect of urbanicity on financial satisfaction; (iii) the global minimum for loneliness is not observed across all conditions; and (iv) for non-white groups, the mean income increases in both urban and rural areas around a minimum located at *d* = 0.009. When we measure urbanicity as population density, we obtain highly similar results in highly urban areas. However, we see differences at intermediate distances versus intermediate population densities: The optima in the three social factors are not reproduced using population density, but a novel U-shape in income is observed.

### Standard deviations

We now address how the variation in responses changes with urbanicity. This variation is quantified in terms of the SD; thus, higher values indicate more spread, while lower values indicate more homogeneity in participants’ self-reports.

**Fig. 3.**
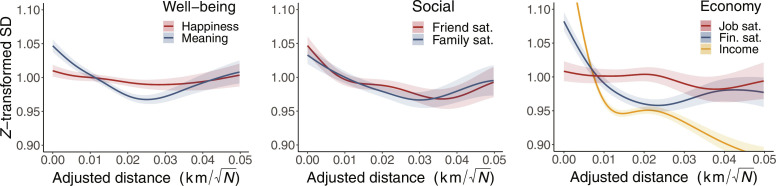
Urban gradients and 95% confidence bands showing how the SDs vary as a function of urbanicity for seven variables grouped along three psychological dimensions. Left: Well-being. Middle: Social satisfaction. Right: Economic satisfaction. Lower values indicate less variation in responses.

The left-most plot in Fig. 3 illustrates the relationship between well-being variables and urbanicity. General happiness remains constant along the urban gradient with minor fluctuations at both ends. Meaningful life follows a “V-shaped” pattern with a minimum at *d* = 0.025 and increased variability in both highly urban and highly rural contexts.

The central plot of Fig. 3 shows how urbanicity relates to variation in the social factors. Both factors display the most variation near city centers and a global minimum between *d* = 0.03 and *d* = 0.04. Beyond *d* = 0.04, the variation in both factors increases as well.

In the right-most figure, we see variations in the three economic factors. We see a strong trend in income where the variation in responses rapidly increases as we approach city centers. Financial satisfaction displays a V-like shape with a global minimum at *d* = 0.02, indicating increased variation in both highly urban and rural areas. For job satisfaction, a small effect with a trend toward increased variation in job satisfaction near city centers.

From fig. S2, we see that the SD results are highly robust with a few exceptions. Similar to what we observed with the mean-level results, excluding London decreases the variability in income responses in cities. In addition, we see increased variability in general happiness when we adjust for participation bias and include cities of at least 50,000 inhabitants. Last, when we operationalize urbanicity as population density, we see some differences between more rural and low population density areas. Rural areas are associated with the least variation in income, while low population density shows substantive variation in income. Low population density areas display the least variation in happiness and meaningful life. This trend is not observed using adjusted distance.

### Optimal distances

**Fig. 4.**
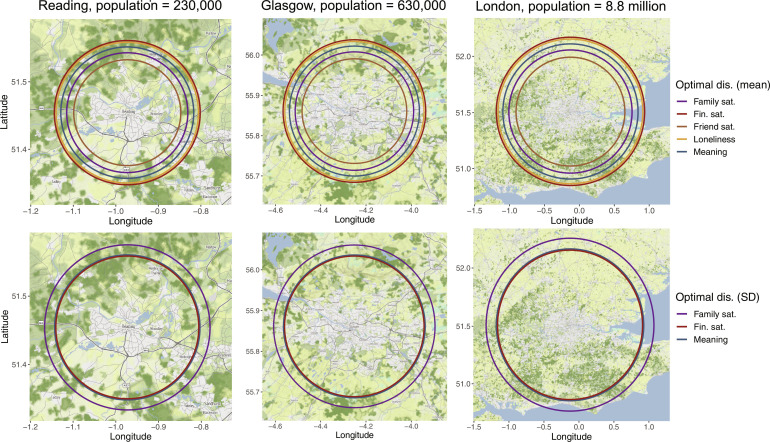
Optimal distances. Distances with optimal means were observed for five variables, and distances with minimal SD were observed for four variables. We show these optimal distances for three cities with different sizes, Reading (230,000), Glasgow (630,000), and London (8.8 million). The top panel shows the radii with the highest average, and the bottom panel shows the radii with the least variation (we omit the optimal SD radius for friendship satisfaction due to its large scale).

Our results also revealed “optimal distances.” By “optimal,” we refer to areas with the highest averages and the least variation. These areas were present for meaningful life, family satisfaction, friendship satisfaction, loneliness, and financial satisfaction. Since the distances are relative to the size of cities, we computed them for three U.K. cities of different sizes. The numeric results are presented in Table 2 for Reading (population of 230,000), Glasgow (population of 630,000), and London (population of 8.8 million). Figure 4 shows that the optimal distances overlaid on geographic maps of the three cities. We see that the distances are located in the hinterlands of cities and that the distances with the most equal responses are located further out than the distances of the highest averages.

**Table 2.** Optimal distances. Our fitted statistical models predicted the highest average satisfaction (means) and the least variance (SD) at intermediate distances between highly urban and rural areas for five variables. The table illustrates where the optimal distances are located for cities with three different populations, Reading (230,000), Glasgow (630,000), and London (8.8 million).

Variable	Healthy peak (mean)	Mean peak (city *N* = 230,000)	Mean peak (city *N* = 630,000)	Mean peak (city *N* = 8.8 million)	Healthy peak (SD)
Happiness	–	–	–	–	–
Meaningful life	0.023 km/√N	11 km	18 km	67 km	0.025 km/√N
Family satisfaction	0.020 km/√N	10 km	17 km	63 km	0.03 km/√N
Friendship satisfaction	0.018 km/√N	9 km	15 km	56 km	0.036 km/√N
Loneliness	0.024 km/√N	12 km	20 km	74 km	–
Income	–	–	–	–	–
Financial satisfaction	0.025 km/√N	12 km	20 km	74 km	0.02 km/sqrt(*N*)
Job satisfaction	–	–	–	–	–

## DISCUSSION

Here, we used a novel and continuous measure of urbanicity to study whether individuals living closest to the urban abundance of social and economic opportunities experience a psychological advantage relative to those living at greater distances. We categorize our results into three classes of phenomena.

The first class pertains to associations between distance and mean levels. In line with agglomeration effects, we find that urban residents have the highest incomes. However, we find no parallel psychological advantages. On the contrary, residents in highly urban areas score worse on all psychological measures covering well-being, social satisfaction, and economic satisfaction. These robust results corroborate existing findings and add dimensions to our understanding of urban psychology. First of all, we replicate the rural happiness paradox observed in economically developed countries where the rural population scores higher on general happiness (*11*–*13*). Second, we show that similar patterns hold for social and economic satisfactions despite the abundance of people, social opportunities, and high incomes in cities. Therefore, our results show that the urban disadvantage is not limited to happiness but also holds for social and economic satisfactions. This suggests a broader conundrum, which we term the “urban desirability paradox,” to highlight the contrast between the popularity of cities and the psychological state of their residents.

The second class of phenomena pertains to the analysis of SDs. We find increasing SDs in all three psychological domains that evidence increased inequality in satisfaction. This relationship is the strongest for income and financial satisfaction, which is in line with existing empirical work that robustly relates economic inequality to urban dynamics (*39*). For friendship and family satisfactions, we observe similar trends with highest satisfaction inequality near city centers. The increased social and economic satisfaction inequality is in line with urban accumulation theories, which document that cities disproportionately benefit the already advantaged (*41*, *43*). On the basis of these considerations, we would expect a similar inequality in well-being. We find an effect of urbanicity on meaningful life but no effect on happiness. This suggests that all variables except happiness follow the predictions of accumulation theories. Why this is the case remains an open question. More generally, it is also an open question why happiness and meaningful life follow a similar mean level trend but differ in terms of SDs.

The last class of phenomena is the optimal distances at intermediate distances between cities and highly rural areas. We found these optimal distances both for means, which indicate the highest averages, and for SDs, which reflect the least variation in responses. We identified optimal distances for five variables, meaningful life, family satisfaction, friendship satisfaction, loneliness, and financial satisfaction. Figure 4 revealed that these radii tend to cluster in the hinterlands of cities. We contend that these results are highly exploratory, and our method does not yield a good uncertainty estimate around the optimal distances. Still, the occurrence across factors has important implications for future research. First, the exploratory findings need independent validation and theoretical understanding. Regarding theory, we can exclude population density as the driver of the optimal social distances since the optima are not reproduced with this measure (see fig. S4 for population density analysis). Second, it shows the importance of using nonlinear methods that can detect effects between cities and rural areas. Failing to do so introduces a confounding variable if these areas are inconsistently classified as urban and rural in studies that rely on simple dichotomies.

We now discuss our findings in light of previous work to assess their generalizability. Our results on well-being agree with (*12*–*14*), which also shows that the urban happiness disadvantage is robust across North America, Western Europe, and large parts of Oceania. The urban economic dissatisfaction is consistent with (*33*) and can be explained by increased living costs, especially housing prices, in cities (*12*). We deem it likely that the economic struggle generalizes as a result of the global “housing crisis” (*44*). Regarding the adverse state of social satisfaction, it is unclear whether it extends beyond the United Kingdom; the existing studies are conflicting (*34*–*36*), and the theoretical drivers are not equivocal with some resources arguing for increased isolation and alienation in urban areas (*15*, *17*), while others have argued for increased opportunities for community building (*22*). Last, we also argued that the observed urban inequality is consistent with the existing literature documenting inequality across economy and health.

We now turn our attention to the limitations of our study, starting with the measure of urbanicity used. This measure is designed as a simple yet nonlinear approach for examining variables in relation to urbanicity and not as a comprehensive definition of urbanicity, as it relies on several simplifying assumptions. For example, city structures are far richer than the strong monocentric form that we assume (*45*, *46*). For our analysis, using one or multiple centers within a city makes little difference because the additional centers are in close proximity relative to the scale at which we compare individuals. However, this argument should be evaluated in the context of specific regions. European and U.K. cities are smaller than in other regions and often revolve around historic centers wherefore the monocentric assumption works well. In contrast, it is often harder to pinpoint a center for American cities that are newer and more sprawled (*46*).

In addition, we stress that distance itself does not cause changes in psychological satisfaction. Thus, our measure does not explain the patterns but diagnoses the state of urban psychology by comparison with more rural settings. The task of explaining these patterns involves mapping out the complex set of drivers that are associated with distance, such as the type of housing, sensory inputs, infrastructure, commuting, types of employment, pace of life, access to education, cultural opportunities, availability of green spaces, and the cost of living. In addition to these place-based factors, the patterns are also shaped by systematic movements of psychologically resilient and vulnerable individuals (*47*). So, the observed optimal distances might arise from happy individuals moving there rather than the locations themselves enhancing individual well-being. Thus, our findings do not imply that moving to or from urban areas will change well-being.

Last, we discuss our measure in relation to population density. By comparing them, we see congruence between densely populated areas and city centers: Both are linked to adverse outcomes across all seven psychological measures. However, low density areas and remote areas do not exhibit similar patterns. Looking at distance, we find optimal distances in the hinterlands of cities for all social satisfaction variables, economic satisfaction, and meaningful life. These are not seen as a function of population density where we find a U-shape in the relationship between population density and income. Future research is needed to understand what areas and mechanics drive these differences.

We acknowledge two main threats to the validity of our three classes of phenomena. First, the U.K. Biobank suffers from a healthy volunteer bias, whereby responders are generally less diverse, better off socioeconomically, and healthier than population estimates suggest (*48*). For this reason, we strongly encourage replications in more diverse samples. However, we have attempted to mitigate the selection bias by following the approach of (*49*), which uses census and population-level data to reduce the bias by 78% using participant weights. From figs. S1 and S2, we see that our results are robust to this adjustment. Furthermore, to assess the relevance of our results across ethnic groups, we reran our results for the following groups: Black and Black British, Asian and Asian British, white, and other. Figures S5 and S6 show little differences between the groups except for income. In the white population, we find increased income in cities, which then decreases and stays constant across the rural gradient. For the three non-white groups, we find a V-shape revealing increased income in cities and then a global minimum at *d* = 0.009, before the average income increases substantially again in rural areas. It is an open interesting question what drives this rural economic advantage for ethnic minorities?

The second threat to our validity is the subjective nature of psychological concepts. To address this, we sought to assess multiple questions with expected similar results. For well-being, we analyzed happiness and meaningful life, and for social satisfaction, loneliness was expected to share trends with friendship and family satisfactions. We were unable to find similar measures of financial satisfaction for comparison. Across well-being and the social domain, we found similar urban gradients for similar questions which increase our confidence in their validity—except for SDs for meaningful life and general happiness, which leaves an open theoretical question. Our confidence is also strengthened by other merits of the study. We study a large sample ranging from 41,000 to 154,000 participants. Rich geographical information allows us to move beyond simple rural-urban dichotomies and distinguish potential sub- and peri-urban effects. Our robustness analysis shows that key findings withstand controlling for age and income, including cities down to 50,000 inhabitants, alternative city size definitions, adjusting for participation bias, defining urbanicity as population density, excluding London–based individuals, and running separate analyses per ethnic group.

A central limitation of our study is the lack of individuals aged 39 and younger. New research that includes this demographic group is called for, given the escalating mental health concerns within this demographic (*50*) and considering our narrative of an urban promise that is most relevant during the formative years. While some of the previous literature, especially on the urban happiness disadvantage, includes younger generations, further research is needed to establish how social satisfaction and economic satisfaction are associated with younger generations.

In conclusion, our results provide evidence that within the 40-plus U.K. population, more urban residents experience the lowest and most unequal levels of well-being, social satisfaction, and economic satisfaction. We also identify areas near cities, yet beyond their boundaries, where the highest and most equal levels of psychological satisfaction are found. These findings carry practical and methodological implications. Methodologically, our study contributes to the growing chorus advocating against simplistic urban-rural dichotomies in urban research (*14*, *37*). Suburban and peri-urban areas have distinct characteristics that set them apart from highly urbanized and rural regions, necessitating the use of granular measures of urbanicity, such as ours. Practically, our study raises a concern for the psychological health of the 56 million Britons residing in urban areas (*51*) and invites further study into the possibility of geographically focused health and policy interventions targeting psychological health. Moreover, by aligning with existing literature on well-being, global inequality, and global housing crisis, we hypothesize that the urban psychological struggles we identified are likely applicable beyond the United Kingdom. Thus, potential city migrants, planners, and policy-makers would do well to acknowledge and tackle the schism that our socially, economically, and culturally most abundant places at the same time exhibit the lowest and most unequal levels of social satisfaction, economic satisfaction, and well-being.

## MATERIALS AND METHODS

We investigated our research questions using the U.K. Biobank. This ambitious data project invited all legally registered U.K. individuals between 40 and 69 years to one of the U.K. Biobank’s 21 assessment centers in England, Wales, and Scotland between 2006 and 2010. The entire dataset covers around 500,000 participants of which we used 156,000 participants with both rich geographical and psychological information (the effective sample sizes are described in Table 1). The study protocol is described in detail (www.ukbiobank.ac.uk/media/gnkeyh2q/study-rationale.pdf), and information on ethical approval given by the North West Multi-centre Research Ethics Committee is found at www.ukbiobank.ac.uk/learn-more-about-uk-biobank/about-us/ethics.

### Variables

Our three main psychological dimensions are well-being, social satisfaction, and economic satisfaction. Within each dimension, we analyzed two, three, and three variables, respectively. The eight variables of interest are presented in Table 1, including the phrasing of the question, sample size, and response type for each item. Histograms of responses for all variables can be found in fig. S3. Participants indicating “jobless” were filtered out of the job satisfaction data. For the meaningful life variable, we discarded “do not know” responses. Income was measured, in British pounds, as the average total household income before tax in five intervals: (i) less than £18,000, (ii) £18,000 to £31,000, (iii) £31,000 to £51,000, (iv) £51,000 to £100,000, and (v) above £100,000. The scores were recoded into single numbers: £15,000, £25,000, £40,000, £75,000, and £130,000. Last, all variables were transformed into *z* scores, which allow for cross-scale comparisons and direct readings of effect sizes in terms of Cohen’s *d*.

### Spatial analysis

All data processing, analyses, and visualizations were conducted in R (*52*). All code is available at our online repository. To measure urbanicity, we calculated each participant’s city size-adjusted distance to the nearest city center. We used the raster spatial analysis package to determine these distances based on each participant’s coarse-grained home coordinates, as well as official city center and city population data obtained from the National Geospatial-Intelligence Agency. These data are also available at our online repository. Specifically, for each participant *i*, we computed the distance to each city *c* and divided this distance by the square root of the city population. The rationale for this normalization is found in (*53*). We also validate this method in a robustness analyzes where we normalize by cities’ spatial size (described further below). After determining each participant’s size-adjusted distance to each city, we retained the lowest value. Since we retain the lowest value, participants are assigned to the nearest city. Thus, if a participant lives 55-km west of London, in the center of Reading, they will not be assigned to London but to Reading. As a result, if a person scores low on our measure of urbanicity, then this implies that they do not live close to any city center. This vector of adjusted distances *d_i_* constitutes our independent variable for all analyses. We give the equation and spatial intuitions for the measure of urbanicity in Fig. 1.

### Statistical analysis

We used generalized additive location and scale models (GAMLSS models) to analyze the relationship between urbanicity and psychological variables. GAMLSS models have the advantage of detecting nonlinear trends and can be fitted in R using the gamlss package (*54*). Following the recommended guidelines of the package, we used the penalized beta splines as the additive component [cf. documentation for the cs() function accessed October 2023]. To estimate the models, we used the Rigby and Stasinopoulos algorithm with five attempts, resorting to the Cole and Green algorithm for up to 60 iterations if convergence was unsuccessful. To achieve a smooth fit and focus on general trends rather than small fluctuations, we controlled the smoothness of the model by constraining the maximum degrees of freedom to 3 for both scale and location. Note that among our dependent variables, six were measured on ordinal scales, one was binary, and one was measured in intervals. For the binary variable—loneliness—we omitted reporting the effect of SD separately since its variance is perfectly related to its mean.

After model estimation, we used the ggeffects package to extract predicted values and confidence bands for both the mean and SD (*55*). We report our results through visualization of the effects and confidence bands. We report and visualize results in the range *d* < 0.05 since we have limited data beyond that. All models are estimated including participants with *d* < 0.07 to improve estimation at 0.05.

In addition, we explored the location of the “optimal distances” after several models revealed optima at intermediate distances between cities and highly rural areas. We computed the exact distances by identifying the location of the maximum through the method of differences. We then quantified the change in our outcome variable—a sort of effect size—by computing the difference in the *z* score between the values at city centers and their maximum values.

### Robustness checks

Our empirical analysis involved several decisions that could affect our results. To assess the robustness of our findings, we varied five types of analytical choices. These are described below.

#### Minimum size

An important choice when computing distances is which city centers to include. On the one hand, including city centers of smaller cities means that we can generalize to a wider range of cities. On the other hand, there may be potential differences between small, medium, and larger cities that we risk overlooking. To navigate this dilemma, we conducted our analysis four times, each time including city centers from cities with populations of at least 50,000, 100,000, 150,000, and 200,000 inhabitants.

#### Robustness to London inclusion/exclusion

Since London is an order of magnitude larger than most other cities, it has a disproportionate impact on the results, especially for low values of our adjusted distance measure. In addition, the urban dynamics of London might differ from other cities due to its more international profile. For these reasons, the main analyses were repeated with all participants assigned to London excluded.

#### Robustness to age and income

The main analyses were repeated with age and income included as independent variables for both means and variances in the estimation of the GAMLSS models. The degrees of freedom for the two covariates were unrestricted.

#### Alternative city size

Last, we checked the robustness of results to alternative definitions of city size. In our analysis, we standardized city size using the square root of population sizes. As an alternative, we obtained geographic areas for the 29 largest cities and computed the radius for each, assuming that they are perfectly spherical. We then repeated the analysis normalizing by the radius rather than the square root of population size.

#### Participation bias

The U.K. Biobank is known to exhibit a healthy volunteer bias (*48*). To address this, Alten et al. (*49*) developed participant weights to correct for participation bias. The weights are obtained by comparing the U.K. Biobank against U.K. Census microdata, considering variables such as year of birth, sex, ethnicity, educational attainment, employment status, regional residence, dwelling tenure, number of cars in the household, self-reported health, and single-person household status. We obtained weights for 151.649 participants, for which we reran the GAMLSS model including the weights.

#### Alternative urbanicity measure

Consistent findings across measures strengthen our belief in their validity. Thus, we compare our results to the alternative measure of population density using 2009 Local Area District population densities (LADs) from the U.K. National Office for Statistics. Participants are mapped onto LADs based on home locations and assigned a surrounding population density. Population densities are highly skewed, with a few London LADs having population densities being an order of magnitude above all other LADs. To remedy this, we log-transformed the population densities. After log transformation, we removed a few low-density outliers with disproportional influence on results. This decreased the sample size from 157.214 to 151.589. In this sample, adjusted distance and population density correlate −0.52. We reran all statistical analyses with the same specification as the main analysis using the population density measure.

#### Robustness across ethnic groups

The concentration of minority groups contributes to the hallmark diversity of cities. Unfortunately, these urban demographics are underrepresented in the U.K. Biobank. We reran our analysis across different ethnic groups to assess to what extent our results generalize across these subgroups. The limited presence of ethnic minorities in rural areas complicates this. To achieve sufficient sample sizes across the groups, we used the broadest ethnic classifications available in the U.K. Biobank. These groups are Black and Black British (*n* = 1130), Asian and Asian British (*n* = 1305), white (*n* = 136,786), and other (*n* = 1963). Because of the rural sparsity of minorities beyond *d* = 0.03, we only present results in the range 0 to 0.03 (all other results are shown in the range 0 to 0.05).
