# Supplementary material for: The urban desirability paradox: U.K. urban-rural differences in well-being, social satisfaction, and economic satisfaction
Source: Sci Adv. 2024 Jul 19;10(29):eadn1636. doi: 10.1126/sciadv.adn1636 (PMC11259172; doi:10.1126/sciadv.adn1636)
Supplement: Supplementary file 1 — Figs. S1 to S6 [file sciadv.adn1636_sm.pdf]

Science Advances
AAAS

Supplementary Materials for

The urban desirability paradox: U.K. urban-ruraldifferences in well-being, social satisfaction, and economic satisfaction

Adam Finnemann *et al.*

Corresponding author: Adam Finnemann, adam.finnemann@gmail.com

Sci. Adv. **10**, eadn1636 (2024)
DOI: 10.1126/sciadv.adn1636

This PDF file includes:

Figs. S1 to S6

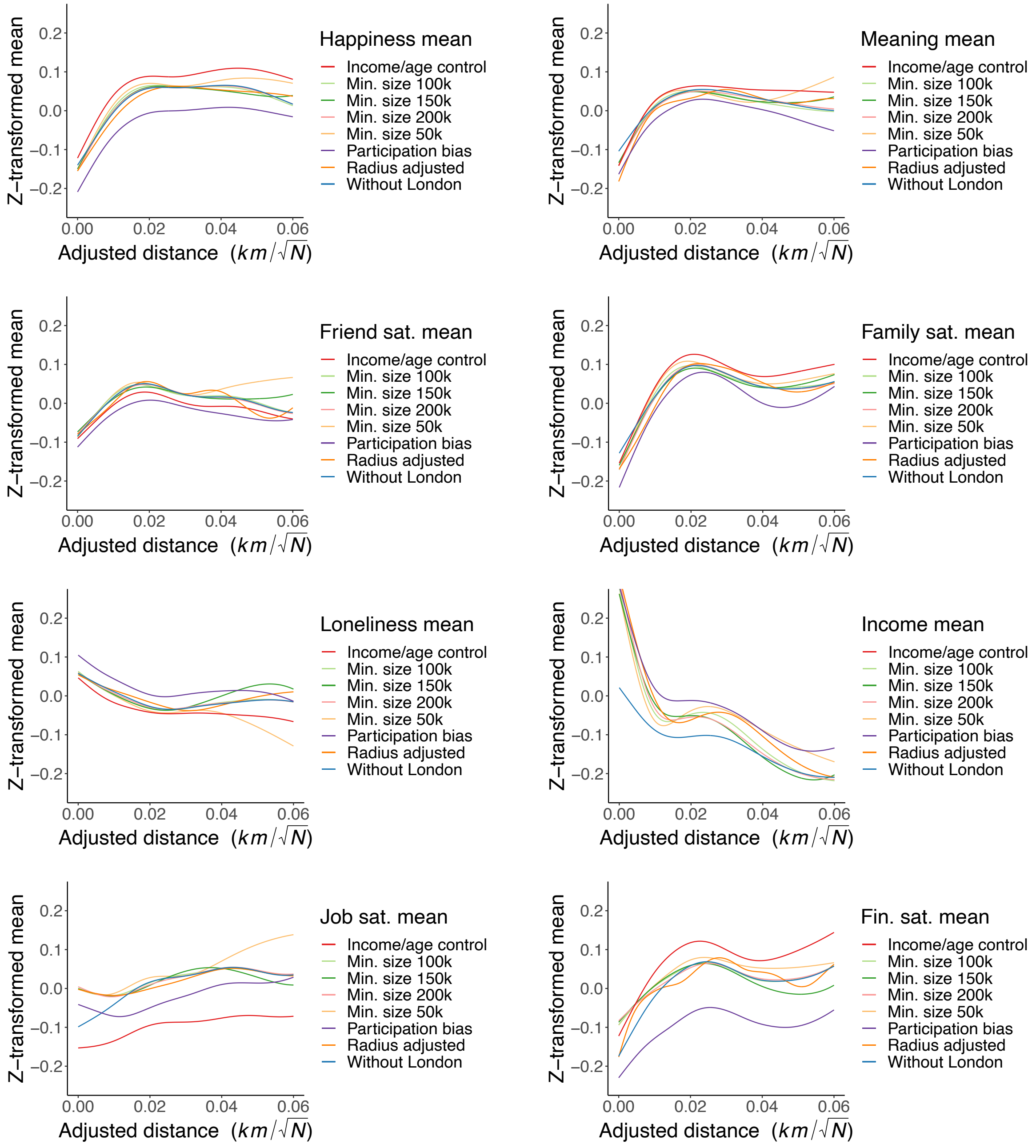

Fig. S1 **Robustness analyses for all mean level results**. Varying centers: adding random noise to city center locations. Min. size: varying the minimum inclusion size for cities, 50k, 100k, 150k, and 200k (same as figures in main). Income/age control: results with income and age added as control variables in the gamlss regression. Without London: all participants assigned to London excluded. City assigned: Participants are assigned to cities based on their raw distance rather than the distance divided by the square roof the population.

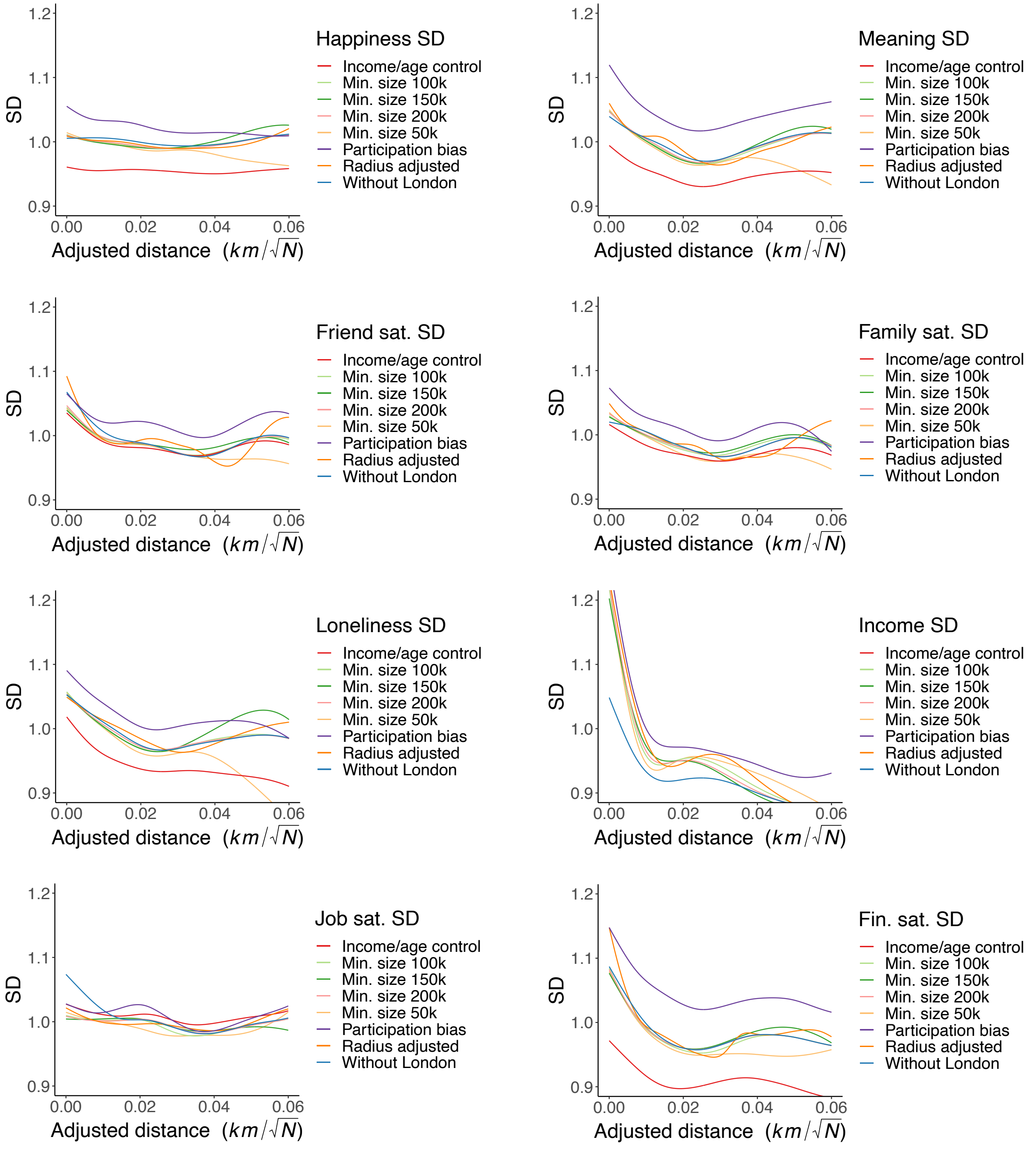

Fig. S2 **Robustness analyses for all SD level results**. Varying centers: adding random noise to city center locations. Min. size: varying the minimum inclusion size for cities, 50k, 100k, 150k, and 200k (same as figures in main). Income/age control: results with income and age added as control variables in the gamlss regression. Without London: all participants assigned to London excluded. City assigned: Participants are assigned to cities based on their raw distance rather than the distance divided by the square roof the population.

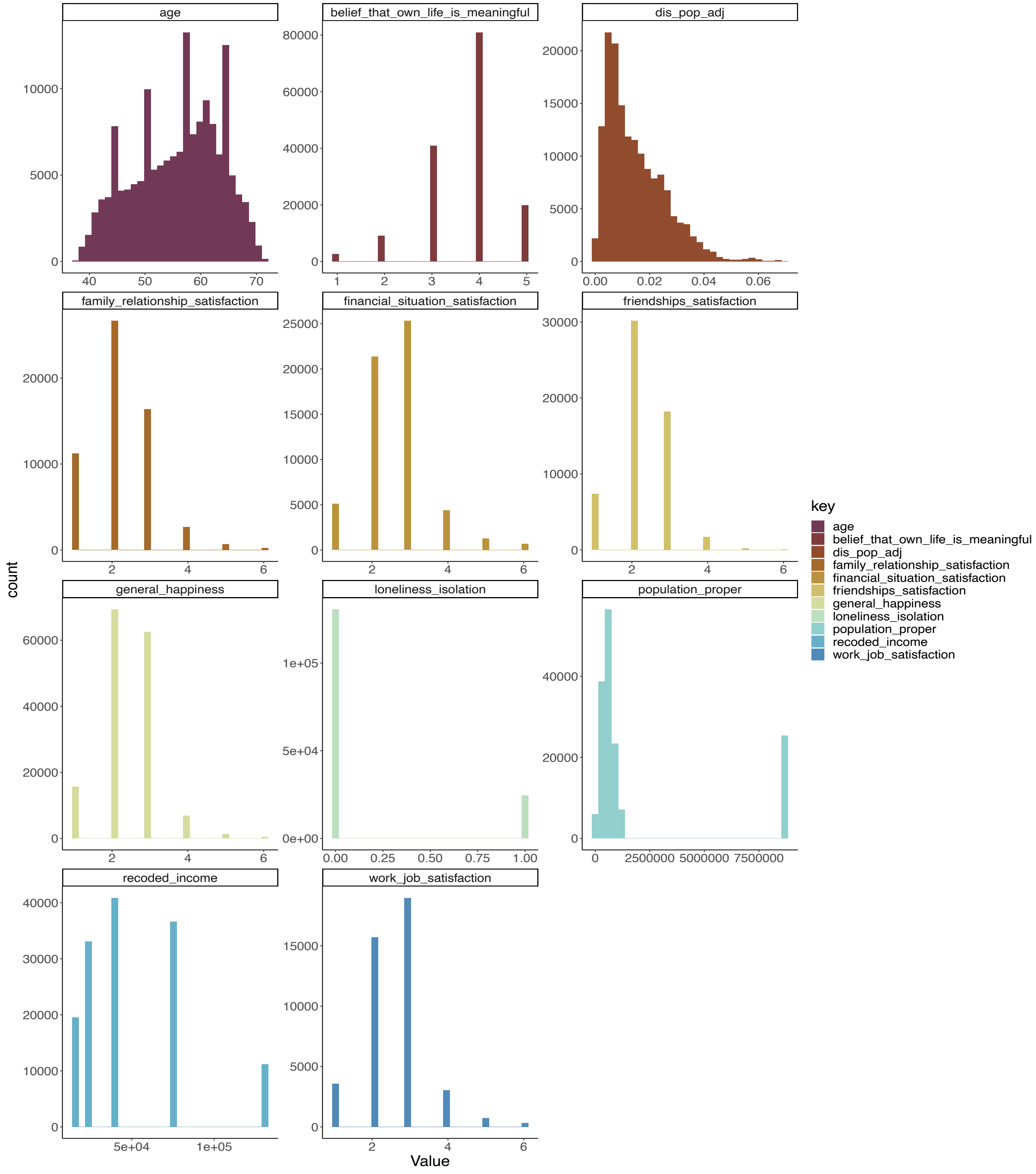

Fig. S3 **Histograms for variables included in the study**.

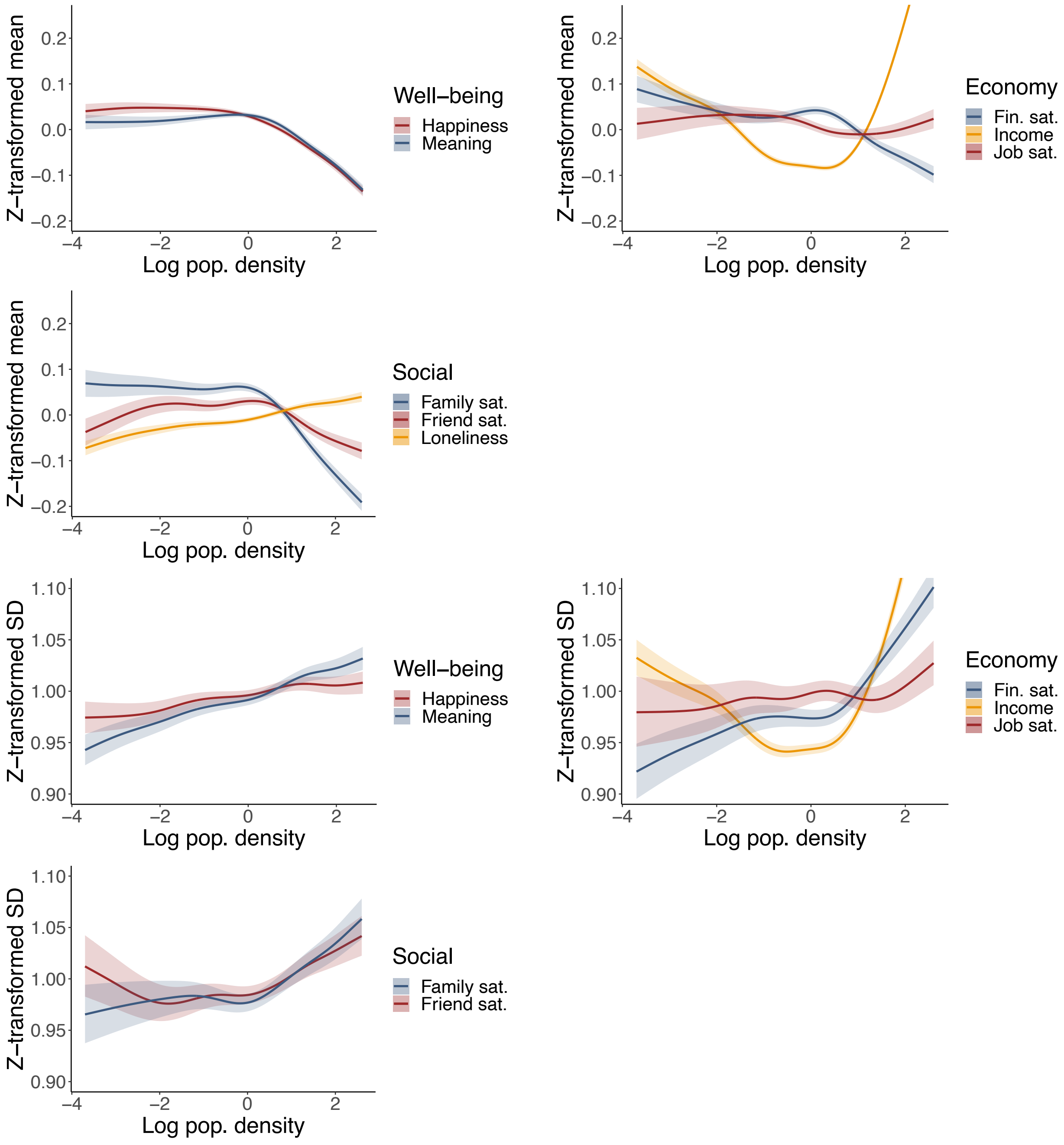

Fig. S4 **All main analyses repeated with an alternative measure of urbanicity in form of logarithmically transformed population densities**.

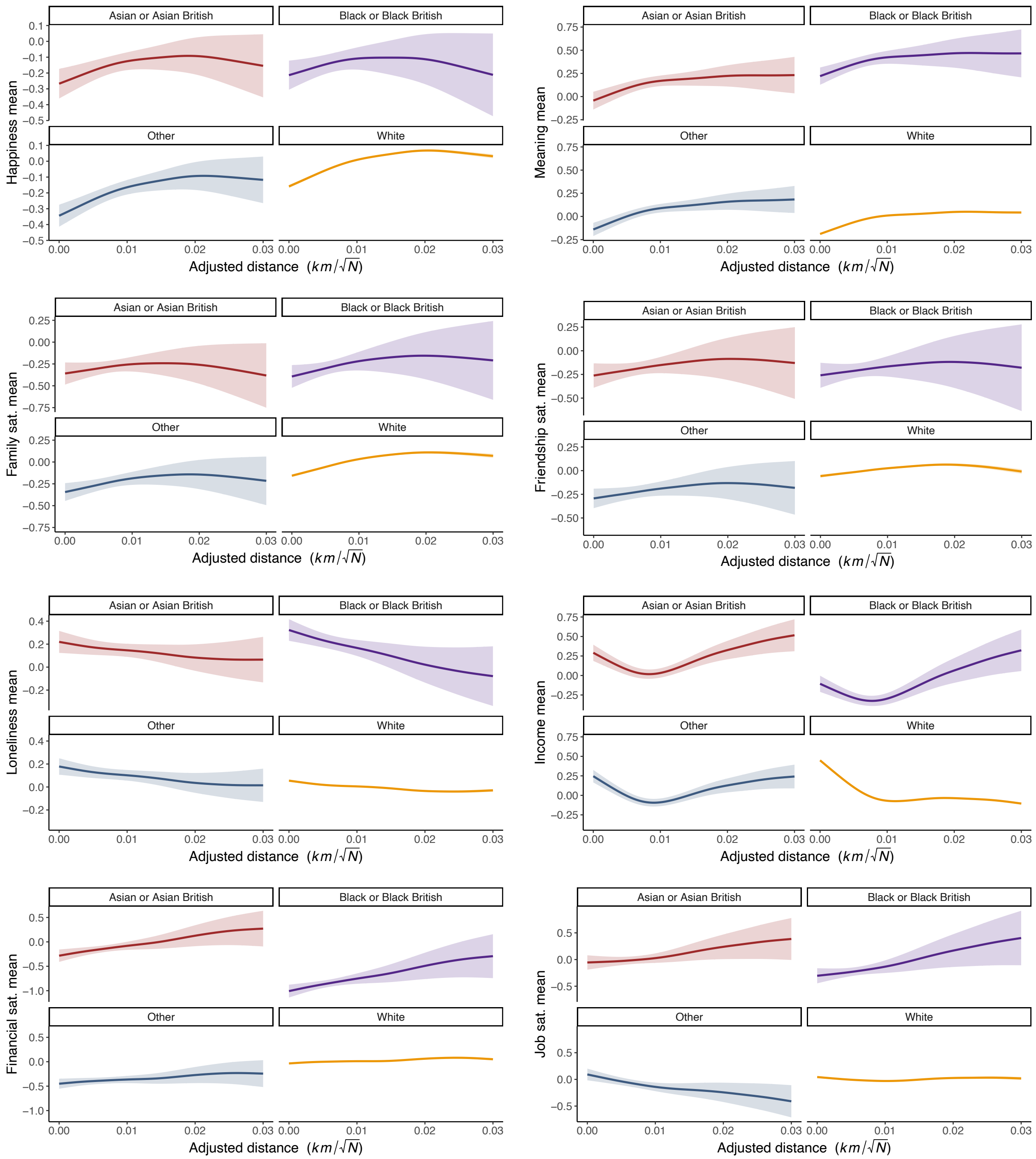

Fig. S5 **Mean results for all eight variables run for four ethnic groups: Asian and Asian British, Black and Black British, other, and white.**

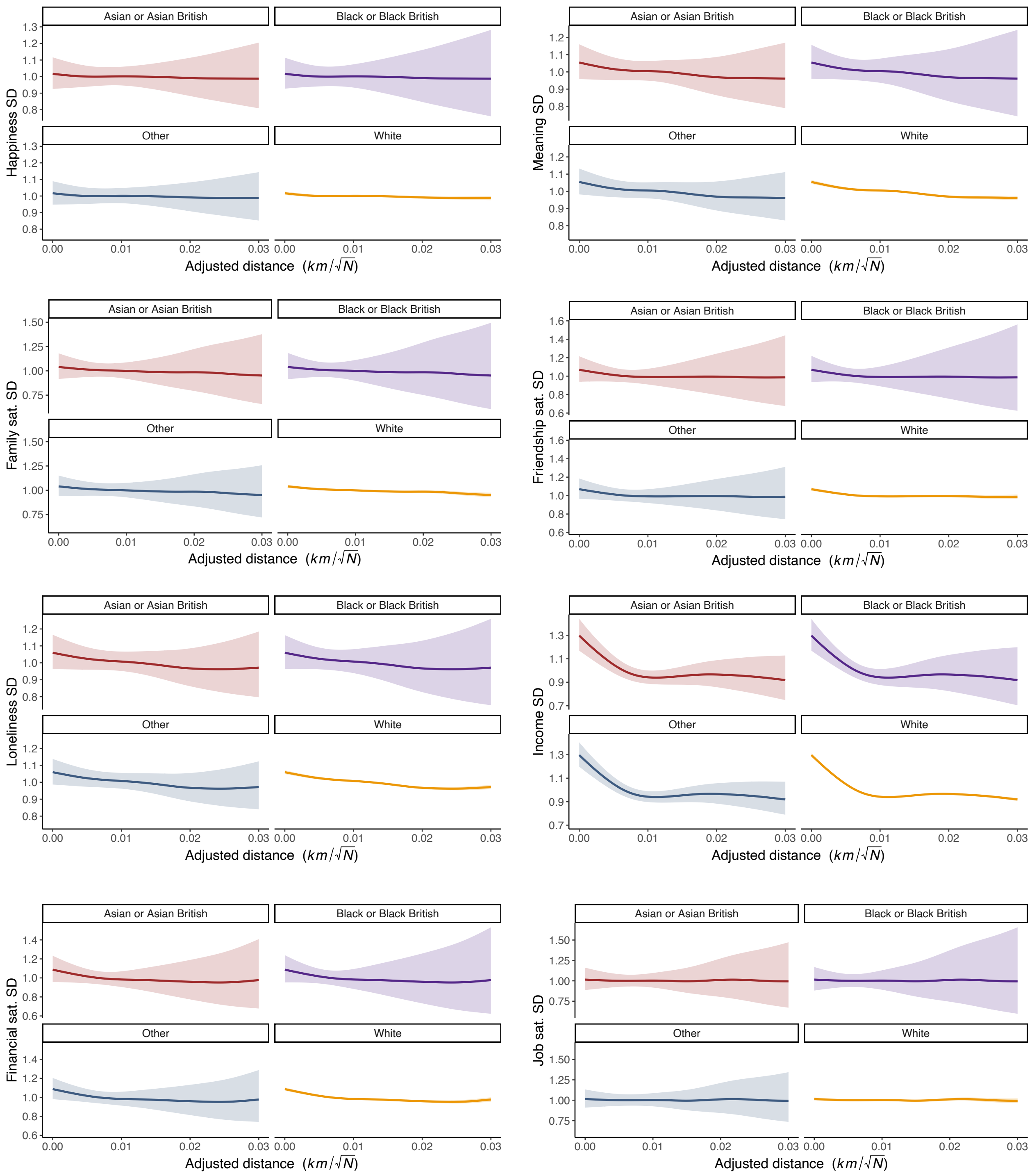

Fig. S6 **Mean resuilts for all eight variables run for for four ethnic groups: Asian and Asian British, Black and Black British, other, and white.**
